# A Foreign Body in the Cervix after Spontaneous Abortion: A Rare Case of a Traumatic Fetal Decapitation

**DOI:** 10.1155/2014/327836

**Published:** 2014-08-10

**Authors:** Danielle Holland, Johnathan Sheele

**Affiliations:** ^1^Eastern Virginia Medical School, 604 Fairfax Avenue Norfolk, VA 23507, USA; ^2^Department of Emergency Medicine, University Hospitals Case Medical Center, Case Western Reserve University, 11100 Euclid Avenue Bolwell 3700, Mailstop BHC5064, Cleveland, OH 44106, USA

## Abstract

Although incomplete spontaneous abortions are common in early pregnancy, fetal decapitation does not specifically appear in the medical literature as a known complication of spontaneous abortion. We present a rare and unusual case of an incomplete spontaneous abortion occurring at home with the mother presenting to the emergency department (ED) with a decapitated fetus and a retained fetal head in the cervical os.

## 1. Introduction

Spontaneous abortion is defined as a loss of pregnancy before 20-week gestation and can affect up to 20% of clinically recognized pregnancies [1]. A completed abortion is clinically diagnosed when all products of conception (POC) are expelled, the uterus is contracted, and the cervical os is closed [2]. In pregnancies of greater than 12-week gestation, the POC, including fetal membranes and fetal or placental tissue, may be retained in the vagina or cervical os, resulting in an incomplete abortion [1]. Cervical evaluation alone has been found to be unreliable in distinguishing between complete and incomplete abortion. In a study of women diagnosed with spontaneous first-trimester abortion, Wong et al. found that 14 of the 47 (30%) women who had been diagnosed with complete abortion had retained POC [3]. Confirming the diagnosis is important in the management and care for women with suspected miscarriage because treatments differ between complete and incomplete abortions.

The clinical signs of miscarriage can be mistaken for menstrual cycle abnormalities, and spontaneous loss of pregnancy can occur even before a woman is aware of the pregnancy [1]. Vaginal bleeding is a common complication of early pregnancy, occurring in up to 20% of pregnancies during the first trimester. In a study from 2004 on 182 women with threatened miscarriage in early pregnancy, the rate of miscarriage among those with first-trimester bleeding was 15%. Of these, women with recurrent bleeding were more likely to miscarry than women with one bleeding episode [4]. Evaluation by transvaginal or transabdominal ultrasound can help confirm pregnancy status and potentially identify abnormalities during pregnancy.

To our knowledge fetal decapitation by way of traumatic self-delivery by the mother has not been previously described as a complication of a spontaneous abortion. However, there are reports of fetal decapitation from vacuum-assisted deliveries, destructive operations, and amniotic band syndrome [5–9].

## 2. Case Presentation

**Figure 1:**
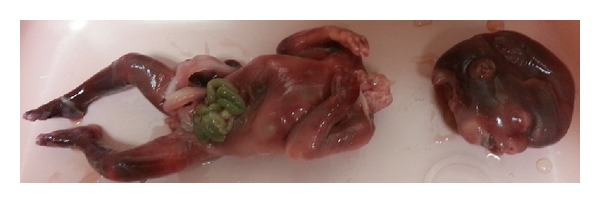
Intrauterine fetal demise with traumatic decapitation after a spontaneous incomplete abortion.

A 26-year-old African American female not known to have ever been pregnant arrived to the emergency department (ED) via ambulance complaining of a one-day history of progressively worsening vaginal bleeding and crampy lower abdominal pain. Prior to her arrival to the ED, the patient stated that she began passing clots, and while sitting down on the toilet, she attempted to pull out a thick vaginal blood clot. Instead, she reportedly grabbed hold of a pair of legs and pulled out a fetus that was missing its head. She dropped the body of the fetus into the toilet and called EMS, who brought her and a decapitated fetus to the hospital (Figure 1).

The last menstrual period was estimated to be at 2 months prior to the date of presentation. The patient reported taking a home pregnancy test about 1 to 2 weeks before her ED visit that was negative. She had a medical history significant for chronic myeloid leukemia, for which she reports taking Tasigna (nilotinib), a tyrosine kinase inhibitor which is FDA pregnancy category D (positive evidence of risk). Her surgical history is significant for ovarian cystectomy and left oophorectomy.

On examination, the patient appeared well and in no distress. Her vitals were stable and physical exam was unremarkable except for mild suprapubic tenderness to palpation. A transvaginal ultrasound was performed, revealing no intrauterine gestation and a markedly thickened endometrium of up to 3.6 cm. Questionable hyperemia of the anterior myometrium and a probable corpus luteal cyst in the right ovary were also noted. A pelvic exam showed a moderate amount of blood in the vaginal vault, along with the fetal head located at the external cervical os. The fetal head was removed using ring forceps, after which the patient was transferred from the ED to the operating room for dilation and curettage and had an uneventful postoperative course.

The pathology report on the fetus reports a 182-gram phenotypic male with the intestinal organs outside of the abdomen cavity through an omphalocele. There was mild ecchymosis, a laceration in the right groin, and the head was disconnected from the body.

## 3. Discussion

Differentiating a complete abortion from an incomplete abortion can be clinically difficult. A complete abortion can be confirmed by visualizing the entire gestational sac, the beta-human chorionic gonadotropin (*β*-HCG) that trend to zero, or a pelvic ultrasound demonstrating an empty uterus after a known intrauterine pregnancy [2]. The cervical os is capable of opening to expel some of the POC and then closing again, causing an incomplete abortion to mimic a complete abortion [2].

Our patient's pelvic ultrasound did not show any intrauterine products of conception and did not identify the decapitated head in the cervical os. Regardless, our patient had an incomplete abortion with the fetal head visualized in the cervical os on physical exam. To our knowledge this is the first reported case of a traumatic decapitation associated with an incomplete abortion complicated by a home delivery. As previously mentioned, the patient reported a recent negative home pregnancy test result and a history of passing clots prior to initial evaluation in the emergency department.

Patients presenting to the ED with signs of a suspected threatened, incomplete, or complete abortion should be evaluated by both transvaginal ultrasound and a pelvic exam to determine if the internal cervical os is open and if any POC are visible. Furthermore, close followup with OB/Gyn should be arranged for threatened and incomplete abortions. Management of first- and second-trimester incomplete abortions includes oral or vaginal prostaglandins, uterotonics, progesterone antagonists, expectant management, and/or surgical dilation and curettage [10]. Additional management options should be considered if there is increased risk of hemorrhage or evidence of infection, or if the woman has had previous adverse or traumatic experience associated with pregnancy [11].
